# COVID-19 Infection and Neuropathological Features

**DOI:** 10.3390/medicines8100059

**Published:** 2021-10-11

**Authors:** Leonardo Freire-de-Lima, Aline Miranda Scovino, Leonardo Marques da Fonseca, Camilla Cristie Barreto Menezes, Carlos Antonio do Nascimento Santos, Marco Edilson Freire de Lima, Debora Decote-Ricardo, Matheus Freire-de-Lima, Kelli Monteiro da Costa, Jhenifer Santos dos Reis, Marcos André Rodrigues da Costa Santos, Celio Geraldo Freire-de-Lima, Alexandre Morrot

**Affiliations:** 1 Instituto de Biofisica Carlos Chagas Filho, Universidade Federal do Rio de Janeiro, Rio de Janeiro 21941-170, Brazil; lfonseca@biof.ufrj.br (L.M.d.F.); cansantos.bio@gmail.com (C.A.d.N.S.); matheusfreiredelima@gmail.com (M.F.-d.-L.); kellimc85@gmail.com (K.M.d.C.); jhnffrrs8@gmail.com (J.S.d.R.); rodrigues8mr@biof.ufrj.br (M.A.R.d.C.S.); celio@biof.ufrj.br (C.G.F.-d.-L.); 2 Instituto de Microbiologia Paulo de Góes, Universidade Federal do Rio de Janeiro, Rio de Janeiro 21941-170, Brazil; ali.scovino@gmail.com; 3 Instituto Oswaldo Cruz, FIOCRUZ, Rio de Janeiro 21040-360, Brazil; ccristie.95@gmail.com; 4 Departamento de Química Orgânica, Instituto de Química, Universidade Federal Rural do Rio de Janeiro, Rio de Janeiro 23890-000, Brazil; marco@ufrrj.br; 5 Departamento de Microbiologia e Imunologia Veterinária, Instituto de Veterinária, Universidade Federal Rural do Rio de Janeiro, Rio de Janeiro 23890-000, Brazil; decotericardo96@gmail.com; 6 Faculdade de Medicina, Universidade Federal do Rio de Janeiro, Rio de Janeiro 21044-020, Brazil

**Keywords:** COVID-19 infection, neuropathology, autoantibodies, neuroinflammation

## Abstract

The pathology associated with COVID-19 infection is progressively being revealed. Recent postmortem assessments have revealed acute airway inflammation as well as diffuse alveolar damage, which bears resemblance to severe acute respiratory syndromes induced by both SARS-CoV and MERS-CoV infections. Although recent papers have highlighted some neuropathologies associated with COVID-19 infection, little is known about this topic of great importance in the area of public health. Here, we discuss how neuroinflammation related to COVID-19 could be triggered by direct viral neuroinvasion and/or cytokine release over the course of the infection.

COVID-19 is a disease caused by the SARS-CoV-2 virus, a beta coronavirus discovered in late 2019 in Wuhan, China. Reports from China at the onset of the outbreak and from other countries thereafter clearly demonstrated that the majority of patients (81%) have mild symptoms without pneumonia or mild pneumonia, and among patients with more significant symptoms, 14% have severe respiratory distress and 5% have respiratory failure, septic shock, and/or multiple organ failure [1,2]. However, today we know with certainty that this virus is capable of infecting several organs and cell types, causing everything from diarrhea and kidney damage to liver, heart, and neurological symptoms [1,3]. Recent work has shown that both neurons and microglia cells express ACE2 and the protease TMPRSS2, essential for cell infection by SARS-CoV-2. In this same work, they were able to detect the presence of viral RNA in 13% of brain samples from patients who died of COVID-19 using qRT-PCR [4]. Another study showed that the virus is capable of infecting 3D human brain organoids or, more precisely, neurons, within 2 days of exposure, causing their death [5]. Additionally, the neuroinvasive potential of SARS-CoV and Middle East respiratory syndrome (MERS)-CoV, which are evolutionarily closely related to SARS-CoV-2, has previously been described [6,7,8]. In addition to these studies that show direct evidence of the presence of the virus in the central nervous system, there are other studies, with a series of epidemiological data pointing to the association of COVID-19 with some neurological disorders, such as anosmia, encephalopathy, stroke, cranial polyneuritis, meningitis, Parkinson’s disease, Alzheimer’s disease, and Guillain–Barré syndrome [9]. Some studies describe the association of Guillain–Barré syndrome with COVID-19. In general, they describe the onset of neurological symptoms after 7 to 10 days of the characteristic respiratory symptoms of COVID-19. The infection by the SARS-CoV-2 virus with the presence of autoantibodies is associated with antigens present in certain tissues, including the brain with high levels of autoantibodies in cerebrospinal fluid that target endothelial, glial, and neuronal epitopes [10]. Some of the antigens described are associated with the central nervous system, such as gangliosides. However, it is not yet known whether infection by SARS-CoV-2 induces the production of autoantibodies against gangliosides in the peripheral nervous system, as occurs with other infections [11,12,13]. A recent work studied the incidence of Guillain–Barré cases among 71,904 COVID-19 patients treated in 61 emergencies in Spain during the two-month peak of the disease. They observed that among patients with COVID-19, the incidence of Guillain–Barré was 9.44/100,000 inhabitants/year, when compared to patients without COVID-19, where the incidence was 0.69/100,000 inhabitants/year [14]. On the other hand, in another study, the authors did not find a significant connection between COVID-19 and Guillain–Barré syndrome. In fact, since there was no increase in incidence for the syndrome in the pandemic period, the incidence in COVID-19 patients actually reduced [15]. The lack of smell (anosmia) and taste (ageusia) that affects almost 60% of patients with COVID-19 seems to be associated with pronounced astrogliosis and microgliosis in the olfactory bulb, probably caused by the virus [4]. It is believed that one of the viral entry pathways is via the neural–mucosal interface by transmucosal entry via regional nervous structures of the olfactory mucosa, or, more precisely, from axonal transport, since the presence of viral RNA and SARS-CoV S proteins was observed in neuroanatomical areas receiving olfactory tract projections. Another respiratory virus capable of invading and infecting the central nervous system through this pathway is the influenza virus [16]. Other viral diseases that affect the upper respiratory airways, such as influenza, damage the olfactory neuroepithelium and can cause smell disorders such as anosmia [17]. Another possibility would be through brain endothelial cells, where they also found immunoreactivity to SARS-CoV S protein. This could even explain anosmia [18]. This neurotropism of the virus by the central nervous system can be intensified by the cytokine storm induced by the infection, which initiates a process of neuroinflammation, inducing an increase in the permeability of the blood–brain barrier [19]. In turn, the neuroinflammatory insult generated may increase the susceptibility to neurodegenerative diseases. This insult to neural cells, such as microglia and astrocytes, can cause an exacerbated release of more inflammatory cytokines and ATP. This activates P2X7 receptors, which in turn can activate the NLRP3 inflammasome pathway within other pathways [20], overshooting inflammation with extensive cytokine release, affecting coagulation, and leading to diffuse lung edema and infiltration by immune cells and inflammatory cytokines and blood–brain barrier disruption. Moreover, disruption of the BBB due to other viral infections has already been proven to trigger long-term development of neurological disorders, such as Alzheimer’s disease, depression, anxiety, and multiple sclerosis [20]. Blood changes due to infection, especially those related to the cerebral endothelium, can affect the coagulation pathways and might be related to cases of stroke related to COVID-19. Other patient reports also suggest that severe SARS-CoV-2 infections are often associated with elevated blood levels of D-dimers and significant platelet reductions, again giving some explanation as to why the patients are at a higher risk of cerebrovascular events in their body [21]. Neurological manifestations due to COVID-19 were also observed through computed tomography. The imaging data show symptoms of necrotizing hemorrhagic encephalopathy. This is a rare disorder leading to brain dysfunction mostly caused by viruses, which results in seizures, liver problems, and mental disorientation following infection. The cascade of cytokines, especially IL-6, causes severe encephalopathy and may even lead to stroke [22]. The presence of higher levels of antibodies against other coronaviruses, which cause the common cold, in the cerebrospinal fluid of patients with Parkinson’s disease suggests a possible involvement of COVID-19 in the pathogenesis of the disease. In addition, the activation of the angiotensin system by SARS-CoV-2, which is related to the pathogenesis of COVID-19, may be important in the neuroinflammatory and neurodegenerative mechanisms observed in Parkinson’s disease [23]. Other viral infections have been associated with the development of transient, or, more rarely, permanent Parkinson’s disease, including influenza A, Epstein–Barr, Japanese encephalitis, Coxsackie, West Nile, Western equine encephalomyelitis, and HIV, mainly due to the induction of neuroinflammation and/or hypoxic brain damage with structural/functional damage within the basal ganglia. In addition, the evidence debated suggests that previous infection with herpes simplex 1, Epstein–Barr, chickenpox zoster, hepatitis C, and influenza A virus may increase the risk of developing Parkinson’s disease in the long term [23]. Contrary to what has been described for Guillain–Barré, thus far only three cases of parkinsonism in the context of SARS-CoV-2 infection have been reported [24,25,26], so there is still no strong association [27].

## Data Availability

Not applicable.
